# Australia’s first women’s mental health hospital

**DOI:** 10.1177/10398562251408228

**Published:** 2026-01-04

**Authors:** Jayashri Kulkarni, Anthony de Castella, Sharon Sherwood, Jess Duda, Adaobe Udechuku, Shalini Arunogiri, Zalie Merrett, Kathryn McKernan, Cynthia White, Eveline Mu, Caroline Gurvich, Sue Williams

**Affiliations:** Cabrini Women’s Mental Health Services, 5267 Cabrini Hospital, Elsternwick, Melbourne, VIC, Australia; HER Centre Australia, Department of Psychiatry, School of Translational Medicine, 2541 Monash University, Melbourne, VIC, Australia; Cabrini Women’s Mental Health Services, 5267 Cabrini Hospital, Elsternwick, Melbourne, VIC, Australia; HER Centre Australia, Department of Psychiatry, School of Translational Medicine, 2541 Monash University, Melbourne, VIC, Australia; Cabrini Women’s Mental Health Services, 5267 Cabrini Hospital, Elsternwick, Melbourne, VIC, Australia; HER Centre Australia, Department of Psychiatry, School of Translational Medicine, 2541 Monash University, Melbourne, VIC, Australia; Cabrini Women’s Mental Health Services, 5267 Cabrini Hospital, Elsternwick, Melbourne, VIC, Australia

**Keywords:** women’s mental health, services, trauma

## Abstract

**Objective:**

To describe the need for specialised women’s mental health services and the development of Australia’s first women’s mental health hospital service in the 21st century.

**Conclusion:**

Women in Australia experience a high level of violence and trauma. Compounding their past experiences with sexual violence, Australian and United States data indicate that a significant percentage of inpatients experience sexual violence during a psychiatric admission. Despite many reviews of mental health service provision, co-gendered wards, with noted incidents of assault against women inpatients, continue to be the usual mode of service in both the public and private healthcare sectors in Australia. Inpatient and community therapeutic programmes have not met the special needs of women. The establishment of Australia’s first women-only mental health hospital at Cabrini Health in 2021 demonstrates the feasibility and value of a specialised, trauma-focused and holistic model of care. These developments highlight the urgent need for broader service reform to ensure safe inpatient facilities and gender-focused therapeutic programs for women.

Despite the growing recognition of the relationship between trauma, violence and mental illness in women; women’s needs for specialised, tailored mental health treatment programs are still largely unmet. Even worse, women who have experienced violence, especially sexual violence, and trauma in their lives can be subjected to further trauma when admitted to co-gendered mental health facilities. We now describe the development of Australia’s first women’s mental health hospital.

## Violence, trauma, & mental illness in women

Violence against women is prevalent in Australia and the world.^1,2^ The United Nations defines violence against women as ‘any act of gender-based violence that results in, or is likely to result in, physical, sexual, or mental harm or suffering to women, including threats of such acts, coercion or arbitrary deprivation of liberty, whether occurring in public or in private life’.^3^ Trauma is an emotional response to violence, a stressful event or prolonged exposure to harm such as abuse, neglect, family violence, or discrimination.^4^ Violence and trauma in early and later life are key drivers in the development of mental illness, particularly in women. Trauma can cause brain biology changes, including heightened hormone sensitivity and altered brain circuitry that drive emotions, cognition and behaviour.

Many women being treated in mental health services have already experienced considerable violence and trauma, including sexual violence. One in five Australian women experience sexual violence, as defined by the World Health Organisation (p. 149).^5^ Sexual violence is associated with physical illness, substance misuse, mental illness, and suicidality.^6^ One in three women presenting to mental health services has experienced domestic violence, including sexual violence.^7–9^

## Women inpatients experience violence & trauma

Compounding their past experiences with sexual violence, Australian and United States (US) data indicate that between 5 and 45% of all inpatients experience sexual violence during a psychiatric admission.^10–12^ Further US data identifies sexual behaviour among inpatients to be between 30 and 70%.^13^ Even when ‘no sex’ policies are explicit, surveys have revealed remarkably high rates of sexual activity within inpatient settings.^6,9,14,15^ Women receiving psychiatric treatment in inpatient settings are at high risk of violence, particularly from male co-patients.^16^

A report on sexual safety in the United Kingdom (UK) mental health wards between 2003 and 2005^17^ identified 122 documented cases, including rape. Even when consensual, sexual relationships beginning during inpatient care, often lead to increased risks for women of longer-term exploitation and violence. In response to escalating assaults on inpatient units, the UK government adopted a strict policy of gender segregation on psychiatric wards.^18^ Although absolute gender segregation has not occurred, UK governments maintain this goal.

In Australia, since the 1990s, we have received numerous recommendations from State and Federal government committees and Royal Commissions into safety for women in mental health systems. Despite the obvious need for tangible solutions, these recommendations have had little impact on the model of inpatient care.

## Women-only areas on public psychiatry wards

Kulkarni and colleagues^19^ campaigned successfully for minor building works to provide a ‘women-only area’ in a public hospital psychiatry inpatient unit. Their follow-up study showed far fewer violent incidents against women^19,20^ in the ‘women-only area’. Most public hospitals in Victoria then developed women-only areas. However, as noted in the Mental Health Complaints Commission’s (MHCC) sexual safety report,^21^ several hospitals breached the women-only areas due to bed shortages or other administrative reasons. The 2018 MHCC report^21^ included data from 90 complaints over 3 years. A clinical and legal team investigated incidents of rape, sexual harassment, stalking and other incidents of violence against women inpatients. A former MHC Commissioner, Dr Lynn Coulson-Barr, stated ‘…one sexual safety incident in a mental health service is one too many. We must never lose sight of the trauma and harm that can be caused by a lack of sexual safety in these environments’.^21^

## Psychiatry in the private health sector

Access to mental health care for women in Victoria remains a significant challenge, particularly within the public system, which supports the most unwell 3% of the population. This leaves a substantial ‘missing middle’, around 30% of individuals, who cannot be managed in primary care settings, yet are not deemed acute enough for public specialist inpatient care. For women, this access gap is further exacerbated by gendered barriers within the healthcare system. Mental health services, excluding perinatal services, both public and private, are co-gendered, despite evidence that women often feel unsafe or retraumatised in mixed-gender environments.

Half of Australia’s psychiatric beds are in the private sector and are co-gendered, with limited safe options for women. An exception is the 2014 Springvale women’s ‘step down’ unit. These systemic shortcomings highlight the urgent need for women-only mental health inpatient units to address gender-specific issues, provide trauma treatment, and bridge the current service gap.

In 2020, during Melbourne’s COVID-19 lockdowns and rising rates of mental illness in women, Kulkarni and de Castella met with the CEO of Cabrini Health to propose a women-only mental health facility. The CEO’s positive response led to 12 months of rapid planning and refurbishment of an existing Cabrini rehabilitation hospital in Hopetoun Street, Elsternwick, Victoria. The development team reviewed programs from 12 private psychiatry inpatient services across Australia, visited Victorian public psychiatry units, consulted UK colleagues and international literature, and visited McLean Hospital in Boston, USA. Input from women with lived experience was provided by the Cabrini Health consumer and carer committee, the MHCC, activist Susan Armstrong, and the Victorian Women’s Mental Health Alliance. Philanthropist Lisa Thurin donated refurbishment funds. Working on major conceptual frameworks plus finer details, such as naming each bedroom after notable Australian women, the multidisciplinary development team, which included women with lived experience, designed and delivered a purpose-built mental health facility, tailored to the needs of women. Considerable work was invested in developing the model of care, the therapeutic environment, and policies, procedures plus governance processes.

Australia’s first inpatient unit for women, named the ‘Lisa Thurin Women’s Health Centre’ (LTWHC), opened on September 1^st^, 2021, with a formal opening on 29^th^ November 2021. Since then, the service has expanded. The inpatient unit continues as the LTWHC, but the broader term that includes the associated community and day programs is ‘Cabrini Women’s Mental Health’.^22^

## The model of care

**Figure 1.**
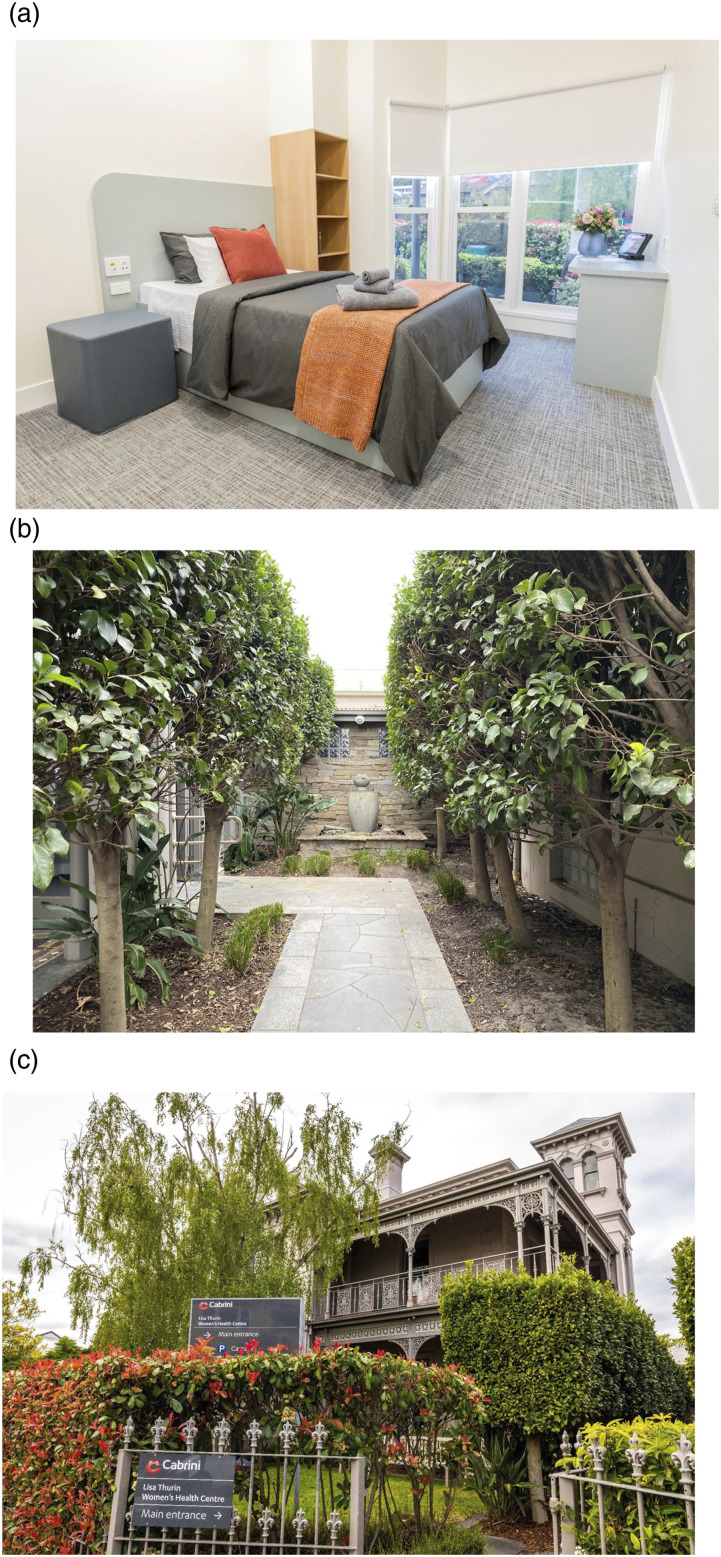
Cabrini Women's Mental Health inpatient unit, known as the Lisa Thurin Women's Health Centre.

The inpatient facility (LTWHC) is situated on a separate site from the main Cabrini Hospital. Its modern bedrooms have ensuite bathrooms and beautiful garden areas (Figure 1). Access to the LTWHC is primarily funded through private health insurance or third-party insurers (Workcover/Department of Veteran’s Affair/Transport Accident Commission), with some women opting to self-fund their care. Recently established, a philanthropic fund enables some women without private health insurance to access care.^22^

Supplemental section contains further details on the model of care.

### The vision

All women who engage with Cabrini Women’s Mental Health will receive care that is personalised to the unique gender aspects of their illness to optimise their recovery and maximise their quality of life.

### Main objectives

- To establish Australia’s first dedicated women’s mental health inpatient facility that caters for the unique treatment and care needs of women with mental illness
- To develop and implement an evidence-based model of care that achieves objectively verified enhanced outcomes for women receiving acute treatment for mental illness
- To provide access and inclusion for women who are lesbian, bisexual, transgender (identifying as female), Aboriginal and/or Torres Strait Islander, all cultures, religions, and ethnicities
- To establish a safe, kind and therapeutic space that supports the unique needs of women requiring inpatient care than community services can provide
- To address the needs of the ‘missing middle’ female population

The guiding principle is that women experiencing significant mental illness will have a better response to treatment and better outcomes when they receive treatment in a safe, women-only facility with treatments and programs that are targeted to meet the specific needs of women and tailored for their specific illness. Holistic treatment is delivered in a gentle, compassionate, validating, kind and collaborative manner with true recognition of violence and trauma in all its forms, plus consideration of the biological factors impacting women’s mental health. Empowering women is a key goal for the whole treatment programme. Consumer and carer representatives provide open and transparent input into the development and delivery of the treatment program, day-to-day operations, and the strategic goals of the service. Consumer feedback is gathered from each woman and utilised to improve care.

### The inpatient programme

We provide care for adult women with mood disorders, complex post-traumatic stress disorder, addiction disorders, and hormone-related mental illness (premenstrual, menopause depression). Most of the women admitted to Cabrini Women's Mental Health Services have complex, interacting symptoms of several diagnoses. Health of the Nation Outcome Scales (HoNOS), Your Experience of Service (YES) surveys, Mental Health Questionnaire 14 item version (MHQ-14), and other data are collected at admission and discharge.

Our psychosocial programme is a mainstay of treatment at LTWHC, plus detailed biopsychosocial assessments and treatments. The program has evidence-based therapies tailored specifically for women. Inpatient stays are 10–14 days; structured within our model of care and continue into community-based therapy. This model is designed to avoid the cycle of repeated hospital admissions and fosters independence by supporting women to reclaim their identity, beyond that of a patient. Upon admission, women are admitted by medical and nursing staff and assigned a care coordinator who works with them during their stay on treatment and recovery goals. The care coordinator supports discharge planning and transition to our community support services.

Our intensive, short-stay inpatient program includes 7 days per week of inpatient therapy provided by multidisciplinary mental health professionals. Each person engages in an individualised programme of at least 6 hours of therapy per day. Therapy is delivered through structured group activities that use treatment modalities such as cognitive behavioural therapy (CBT), dialectical behavioural therapy (DBT), schema therapy and acceptance and commitment therapy (ACT). Eye-movement desensitisation and reprocessing (EMDR) therapy, cognitive processing therapy (CPT) are provided in group and individual formats. We also offer several complementary therapies that support movement and creativity including trauma-informed yoga, exercise programs, art therapy, music therapy and mindfulness.

### The community programme

Our specialised community day programs are designed to meet women’s needs at various stages of their mental health journey. These programs can be accessed independently or to complement the short inpatient stay, creating a coordinated and comprehensive continuum of care. Delivered in groups of 12 or fewer participants, the programs offer a structured and supportive environment where experienced clinicians facilitate therapeutic work. Programs are delivered at a dedicated site located directly across from the inpatient unit, ensuring connection. Lived experience feedback enables program adaptation and expansion, for example, our online programs, which improved accessibility and connection for rural, regional, and interstate women. To further support this continuum, HER Therapy was established as an individualised, low-cost service staffed by an expert, multidisciplinary team. HER Therapy allows for extended engagement for women with complex trauma histories who require longer-term, consistent support.

In February 2025, the service expanded with the introduction of the HER Neurodivergence Program. This program focuses on recognising and responding to the distinct ways in which women present with Autism and Attention Deficit Hyperactivity Disorder. Acknowledging that many women remain undiagnosed or misdiagnosed, often in the context of co-occurring mental health conditions, the program offers collaborative, comprehensive, and holistic assessments. Tailored treatments are provided, ensuring that support is responsive, inclusive, and evidence-based.

### Our workforce

To effectively deliver inpatient and outpatient programs, and to support the ongoing development of our workforce, we ensure that staff receive appropriate, evidence-based training. Staff undertake training in DBT, schema therapy, CPT, ACT, Seeking Safety, and General Psychiatric Management (GPM). Additional training includes introductory education in autism, EMDR, and the Multi-Agency Risk Assessment and Management (MARAM) framework.

We are intentional in our recruitment practices, seeking all genders who are not only clinically skilled but also align with our values, mission, and model of care. We prioritise the recruitment of individuals who are kind and passionate about caring for women clients. Our investment in purposeful recruitment, ongoing training, and robust supervision forms the foundation for delivering safe, consistent, person-centred, trauma–informed care (see Supplementary for details on staffing).

### Integrating spirituality into care

Within the inpatient unit, the Space for Grace program is a distinctive and innovative element of the therapeutic model, offering both group and individual pastoral care that addresses spirituality as a dimension of holistic mental health. Facilitated through group sessions and optional one-to-one sessions, the program provides a safe, inclusive space for reflection and emotional integration. Rooted in the principles of person-centred and trauma-informed care, Space for Grace is not confined to a particular religious tradition. Rather, it embraces a broad, inclusive understanding of spirituality, welcoming individuals from all faiths and belief systems, including those who identify as spiritual but not religious. The program supports individuals in exploring existential themes, loss, identity, and healing through a spiritual lens, which has received excellent feedback from participating women.

### Ethics statement

The Ethics Committee of Alfred Health clarified and approved that patients’ participation in the YES survey was considered as their implied consent for the analysis and publication of this anonymised data.

### Results

In this paper, we focus on feedback data collected from women who were admitted as inpatients and participated in the community programme using an adapted version of the YES survey. We will present more data, in greater detail, in later publications and recognise the broad nature and the limitations of the YES survey.

YES is a national instrument, where mental health consumers accessing a service rate their experience of the service across 26 questions using a 1–5 response scale. Responses are averaged for each question and then calculated as percentages of the total responses. The YES survey used at Cabrini Women’s Mental Health includes additional questions relating to the community programme.

We present YES data, in Table 1, for the time frame of September 1^st^, 2021, to September 1^st^, 2024. 1034 women were admitted during this time; 972 surveys were completed (94%). 922 women from Victoria, 48 from interstate and 4 internationals.

**Table 1.** Your Experience of Service (YES) survey data from patients [details omitted for double-anonymised peer review]

Question	% Positive
Quality of care	
I have been treated with respect and dignity at all times	95%
Overall, the quality of care provided by the hospital has been excellent	93%
I would recommend this hospital to a friend or family member, if they needed psychiatric care	93%
Safety and privacy	
My privacy was respected	97%
The hospital was clean and well maintained	97%
I have felt safe whilst at this hospital	95%
Services were appropriate for my age	93%
I was informed about the cost of my hospital stay and services	91%
My physical health was assessed, and appropriate care was provided when needed	84%
I was able to access the hospital services as soon as I needed to	83%
My concerns and complaints were addressed	76%
Mental health and wellbeing*	
I am more hopeful about my future	79%
I feel I will be better able to deal with crises	76%
My sense of well-being has improved	75%
My symptoms are not bothering me as much	60%
Clinical staff	
I felt welcome at this hospital	97%
Hospital staff were positive that their mental health and quality of life could improve	95%
Hospital staff were available if they needed them	93%
When I had questions, the hospital staff gave helpful answers that I could understand	92%
My individuality and personal preferences were respected	92%
I had opportunities to discuss my progress with the staff caring for me	89%
Staff were sensitive to cultural backgrounds	87%
Hospital staff helped me obtain the information I needed so that I could take charge of managing my illness	87%
I was informed about and encouraged to use self-help or peer support groups in the community	79%
With my permission, my nominated carer was involved in my hospital treatment	76%
Treating psychiatrists	
I have been involved in decisions about my care and treatment	89%
I have been involved in planning the care I may need after I leave the hospital	88%
When I had questions, my treating psychiatrist gave helpful answers I could understand	78%
My treating psychiatrist ensured that I understood the effects of my treatment options	73%
My treating psychiatrist and hospital staff worked as a team in my care and treatment	79%
Community program	
Overall, the quality of care provided by the hospital’s community program services has been excellent	93%
Community program staff were positive that their mental health and quality of life could improve	88%
Community program staff gave helpful answers that they could understand	81%
Community program staff were available if they needed to talk to them	73%
I was able to access the hospital’s community program services as soon as I needed to	88%
I have been involved in planning the care I may need after I complete the community program	50%
Any concerns or complaints I had about the hospital’s community program services were addressed	90%

*After inpatient admission +10 week community programme.

## Discussion

The main rationale for setting up a women’s mental health facility is to deliver mental health care for women in a safe, respectful and kind manner; with programmes and treatments that are tailored to meet women’s needs. Australia’s first women’s mental health hospital, as part of Cabrini Health, called the Lisa Thurin Women's Health Centre (LTWHC) has thus far achieved this important goal. Women reported feeling treated with respect and dignity, would recommend the facility to others, and found the quality of care to be excellent. This endorsement for a women’s mental health service model is important to export to other locations and types of services.

LTWHC has accomplished a great deal in a short space of time, but a major challenge is the recruitment and retention of private psychiatrists with expertise in women’s mental health. Many psychiatrists have expressed their preference for office-based consulting, clinical work or telehealth, not for providing inpatient care. In recent times, primary healthcare practitioners have taken on a growing role in supporting individuals with mental illness and have expressed a strong interest in contributing to the hospital-based care of women with mental health conditions. At the LTWHC, they are valued collaborators and experts in delivering high-quality, holistic inpatient care.

Importantly, the model of care developed by the LTWHC can inform other women’s mental health facilities in Australia. New women’s mental health services are described in Table 2. Ongoing research and evaluation are essential for refining the model of care, guiding best practice and strengthening the case for adoption of women-specific services across Australia. A collegiate network has developed between women’s mental health service providers to assist each other in the development of mental health services for women across our nation.

**Table 2.** Women’s mental health facilities in Australia

Women’s mental health facilities	Type	Year of establishment
Springvale Prevention & Recovery Care Centre (PARC). VIC	‘Step down’ PARC	2014
Sunshine PARC ‘Yanna Yanna’. VIC	‘Step down’ PARC	2022
Women’s Recovery Network (WREN). VIC	Women’s acute inpatient unit & community services. A public and private partnership, including Goulburn Valley area.	2023
Northern Hospital Epping Women’s Mental Health Inpatient Unit. Vic	Women’s mental health inpatient unit. Public mental health inpatient unit	2025
Ramsay Clinic Thirroul. NSW	Women’s inpatient & outpatient service for trauma. Private health	2022
Cockburn Women’s Mental Health Service. WA	Women’s mental health inpatient and community services. Public health facility, part of Fiona Stanley Hospital	2024

There are many media and scholarly commentaries on the fractured state of the mental health system in Victoria and across Australia. A new way forward must involve recognising and meeting the unique mental health needs of women across the lifespan. Women represent more than half of those seeking mental health support, a demand that has intensified in the wake of the COVID-19 pandemic. It is time for women’s mental health to be acknowledged as a national priority. Rebuilding mental health systems must include gender-specific, evidence-based treatments delivered in environments that are safe, compassionate, and responsive to the lived experiences of women. Tailored models of care, including effective trauma therapies, must be embedded within services that are designed for women, reflecting both the diversity and complexity of their mental health journeys. What was once considered an impossible vision of a dedicated, thriving, and effective women’s mental health service with proven outcomes is now achievable and essential. The future of mental health in Australia must include a clear and enduring commitment to specific care that enables women to heal, recover, and flourish.

## Supplemental Material

Supplemental Material - Australia’s first women’s mental health hospital Supplemental Material for Australia’s first women’s mental health hospital by Jayashri Kulkarni, Anthony de Castella, Sharon Sherwood, Jess Duda, Adaobe Udechuku, Shalini Arunogiri, Zalie Merrett in Australasian Psychiatry

## Data Availability

The datasets generated during and/or analysed during the current study are available from the corresponding author on reasonable request.*
