# Supplementary material for: Australia’s first women’s mental health hospital
Source: Australas Psychiatry. 2026 Jan 4;34(3):268–74. doi: 10.1177/10398562251408228 (PMC13222380; doi:10.1177/10398562251408228)
Supplement: Supplemental Material - Australia’s first women’s mental health hospital [file sj-pdf-1-apy-10.1177_10398562251408228.pdf]

Supplementary Material: [details omitted for double-anonymised peer review] **– Model of Care and Staffing**

Model of Care

[details omitted for double-anonymised peer review] delivers a specialised, women-only service designed to provide safe, trauma-informed, and empowering care. The service integrates acute inpatient care with structured day programs, creating a continuous recovery pathway. The inpatient unit is a 30-bed facility delivering a focused 14-day program. Admissions are based on clinical triage, with up to two per year per client and a 3–6-month gap between episodes. The Community Day Program is delivered in half-day blocks to encourage attendance while allowing clients rest and reflection. It provides step-down or direct-entry care in accelerated (10-week) or extended (20–24 week) formats, requiring at least 80% attendance. Programs reinforce skills, provide peer support, and maintain continuity of care.

Care is holistic, addressing mental, emotional, physical, and spiritual wellbeing, with personalised treatment plans, mandatory outcome measures (Health of the Nation Outcome Scales (HoNOS), Mental Health Questionnaire -14 item version (MHQ-14), Patient Experience (PEx), Depression Anxiety Stress Scale – 21 item version (DASS-21), Beck Depression Inventory, Beck Anxiety Inventory), and detailed discharge summaries to support continuity.

Our model of care is explicitly inclusive of all individuals who identify as women. This approach acknowledges the diverse experiences of violence and trauma among women, including those in same-sex relationships and women of diverse gender identities. In practice, this has been implemented successfully, with positive therapeutic experiences reported and no complaints raised from other women in the unit.

Programme Details

Core to the model is intensive group therapy. A typical inpatient day combines structured group therapy, psychoeducation, creative and physical therapies, pastoral support, and skill-building activities. Reflective breaks are incorporated between sessions to promote autonomy, skill consolidation, and self-care. This structured yet flexible approach fosters peer connection, engagement, and sustainable recovery.

During the inpatient stay, women spend six hours per day in various structured group activities that use treatment modalities such as cognitive behavioural therapy (CBT), dialectical behavioural therapy (DBT) and acceptance and commitment therapy (ACT). Eye-movement desensitisation and reprocessing (EMDR) therapy is provided in individual and group formats. We also offer several complementary therapies that support movement and creativity including trauma informed yoga with our exercise physiologists, art therapy, and mindfulness. The inpatient therapy program operates seven days a week and is run by highly trained, multidisciplinary mental healthcare team.

Additional service options support clients with more complex or ongoing needs. The Neurodivergent Assessment Centre can be accessed during inpatient admission or as an outpatient service, providing tailored assessment and support. Women may also engage in

HER Therapy, 1:1 outpatient sessions with a mental health clinician, typically for those requiring more intensive, ongoing therapy. Most women, however, continue to benefit primarily from the day program. Program details are available from the website [details omitted for double-anonymised peer review]

Staffing Profile and Purposeful Recruitment

The service is delivered by a multidisciplinary team including consultant psychiatrists, specialist GPs, registrars, nurses, psychologists, occupational therapists, social workers, creative/physical therapists (art, music, exercise physiology), and pastoral care for spiritual support. Allied health staff coordinate care and deliver therapy across inpatient and day programs. Staff-to-patient ratios are flexibly adjusted according to occupancy, acuity, and program activity.

Staff recruitment is purposeful; selecting candidates with prior known excellence for working in mental health facilities and alignment with the unit's culture and values. Staff are evaluated for clinical competence, collaborative style, and ability to contribute to a safe, supportive, and empowering environment. All team members receive program-specific training and trauma-informed communication guidance to ensure consistent, recovery-oriented care.
